# CLINICAL FEATURES AND OUTCOME OF INFECTIVE ENDOCARDITIS IN YEMENI PATIENTS TREATED WITH EMPIRICAL ANTIBIOTIC THERAPY

**Published:** 2010

**Authors:** Al-Aghbari Khaled, Alezzy Yahya Al-Noami, Mohamed Al-Ansi, Ahmed Askar Faiza

**Affiliations:** *Department of Internal Medicine, Faculty of Medicine, Sana’a University, Yemen*

**Keywords:** Infective, Bacterial, Endocarditis, Echocardiogram, Blood culture, Vegetations

## Abstract

**Background:**

Infective endocarditis is a common disease in Yemen. Although the incidence of rheumatic valvular disease and uncorrected congenital heart disease in adults is high in Yemen, there are few data regarding the pattern, characteristic features and outcome of infective endocarditis in Yemen.

**Objective:**

The aim was to study the characteristic, clinical features and diagnostic criteria of infective endocarditis in Yemeni patients and the outcome in patients treated with medical therapy.

**Patients and Method:**

Seventy-two consecutive patients admitted to Kuwait teaching hospital in Sana’a with suspected infective endocarditis between June 1, 2005 and June 1, 2007 were included in this study. A questionnaire including history, clinical findings, and result of requested investigations, treatment, complications and outcome was used. The diagnosis was based on Dukes criteria, which proposed two major or five minor criteria. In our study, we included raised erythrocyte sedimentation rate (ESR) as a minor criteria. The patients were classified as definite, possible and rejected cases. All patients received empirical antibiotic therapy.

**Results:**

The mean age was 28.56 ± 14.5 years. Men were 30 (42.2%) while women were 42 (57.7%). 59 (81.9%) of the patients had been admitted due to fever. Past history of rheumatic heart disease was positive in 38 (53.3%) of the patients. Mitral regurgitation was the commonest form of valvular affection 54 (82.1%) of patients followed by aortic regurgitation in 45 (63%). Anemia was present in 53 (74%) while raised erythrocyte sedimentation rate was high in all patients. Vegetations were detected by transthorasic echocardiography in 51 (70.83%) of the patients while blood culture was positive in only 7 (9.6%). After adding high ESR to the criteria, definite infective endocarditis were found in 34 (47.2%); possible in 38 (52.7%) and no rejected patients. Sixty-two (87.5%) of the patients improved and were discharged in good general condition. Nine patients died, an In hospital mortality of 12.5 %.

**Conclusion:**

patients with IE in Yemen were younger than those patients in western countries, RHD is the commonest predisposing factor. Duke’s criteria in addition to high ESR as minor criteria improved the diagnostic possibilities and using empirical antibiotic therapy improves the outcome.

## Introduction

Infective endocarditis (IE) is one of the most feared complications of structural heart lesions. Early referral, high yield of blood cultures, and appropriate use of antibiotics has decreased the morbidity and mortality of this disease in the developed world^1^,^2^. In the developing world like Yemen where rheumatic heart disease (RHD) still common, antibiotic prophylaxis for potential septic procedures is not routine and late referral is common. It remains an important cause of morbidity and mortality in all age groups^3^,^4^. During our daily practice in Yemen, we deal with advanced cardiac lesions beyond surgical correction, usually in young age. Positive blood culture in suspected cases of IE is low. This circumstance encouraged us to study the characteristics of Yemeni patients who presented with a suspected diagnosis of IE. Hoping to improve the diagnostic possibilities, we suggest to add an elevated ESR as minor criteria which is commonly elevated in such situation.

## Patients and methods

This study was conducted from June 1, 2006 to May 30, 2007. All cardiac patients who presented to our hospital with suspected infective endocarditis were included. Revised Duke’s criteria (Table 1) was used for the diagnosis of IE.

**Table 1:** Duke Criteria for the diagnosis of IE

Major criteria *Positive blood culture:* Typical microorganism for IE from two separate blood cultures Or Persistently positive blood culture, defined as recovery of a microorganism consistent with IE from: Blood cultures drawn more than 12 hr apart Or All of three or a majority of four or more separate blood cultures, with first and last drawn at least 1 hr apart Positive echocardiogram: Oscillating intracardiac mass, on valve or supporting structures, or in the path of regurgitated jets, or on implanted material, in the absence of an alternative anatomical explanation, Or Abscess Or New partial dehiscence of prosthetic valve, Or New valvular regurgitation (increase or change in preexisting murmur not sufficient) Minor criteria *Predisposition:* Predisposing heart condition or intravenous drug use Fever 38.0°C (100.4°F) Vascular phenomena: major arterial emboli, septic pulmonary infarcts, mycotic aneurysm, intracranial hemorrhage, conjunctival hemorrhages, Janeway’s lesions Immunological phenomena: glomerulonephritis, Osler’s nodes, Roth’s spots, rheumatoid factor Microbiological evidence: positive blood culture but not meeting major criterion as noted previously. Serologic evidence of active infection with organism consistent with IE Echocardiogram: consistent with IE but not meeting major criterion
Diagnosis of IE: *Definite:* Two major criteria One major + three minor criteria Five minor criteria *Possible* One major + one minor criteria Three minor criteria.
Rejected: Firm alternative diagnosis for manifestations of endocarditis Or sustained resolution of manifestation of IE with antibiotic therapy for 4 days or less, Or No pathological evidence of IE at surgery or autopsy, after antibiotic therapy for 4 days or less.

In addition to Duke Criteria, we included raised ESR as minor criteria in the absence of an extra cardiac infection. Examination and clinical findings were recorded and all patients had three sets of blood culture. In patients who were receiving antibiotics before admission, blood samples for culture were taken after stopping the antibiotics for at least 48 hours.

The culture was considered typical if classical organism like streptococci or enterococci or HACEK group were found. A single isolation of coagulase negative Staphylococci were considered a contamination.

Other blood test like hemoglobin (Hb), hematocrit value (HCT), total WBC, differential blood count, ESR, electrolytes, renal function, Rheumatoid Factor (RF), liver function test, serology were requested when necessary and indicated. Urine for microscopic hematuria and Chest X-ray were routinely requested for all patients.

Only one experienced operator in transthoracic echocardiography performs the echocardiography. Vegetations were defined as typical if a mobile dense mass was found attached to the valve or their supporting structure. This mass should be in the path of turbulent jets of blood passing through regurgitation or stenotic valve or intra cardiac shunt. Suspicious echo cardiac densities which were not meeting the major criteria were classified as minor criteria.

Abdominal ultrasound was done when complications such as renal, splenic infarction, or abscess were suspected. CT for brain was performed when neurological complications occurred. When diagnosis was made, empirical antibiotics were started (penicillin group 2-3 million I.V every 4 hours + Gentamicin; 1mg/kg q 8hours for the first two weeks and then penicillin for the last two weeks or ceftriaxone 2g I.V. for 28 days.

Dosage and duration of antibiotics, outcome of medical therapy, complications, indication for surgery and its results were recorded.

## Results

Seventy two patients were included with a mean age 28.56±14.5 (minimum age was 12 years and maximum age was 60 years). 58.7% (42 patients) were women and 41.6% (30 patients) were men. Thirty three (46%) of the patients came from Sana’a city and 54% from outside Sana’a.

Most of the patients admitted because of prolonged fever more than one week(59) 81.9%. Other presenting symptoms and baseline characteristic are shown in Table (2)

**Table 2:** Baseline Characteristic Of The IE Patients (n=72)

Baseline characteristics	No. of patients	%
**Mean age**	28.56+/-14.5	
Male gender	30	42.2
History of RHD:	38	53.3
MR	54	82.1
MS	40	55.6
AR	45	63
AS	4	5.6
Double valves (mitral and Aortic regurge)	38	53.3
MVP	2	2.8
**Congenital heart disease:**		
VSD	1	1.4
PS	1	1.4
**Symptoms:**		
Fever	59	81.94
Shortness of breath	37	51.39
Cough	15	20.83
Sweating	14	19.44
Arthralgia	2	2.78
Wasting	14	19.44
Pallor	24	33.33
Stroke	6	08.33
Hepatomegaly	22	31
Splenomegaly	11	15.4
Hepatosplenomegaly	3	4.2
Splinter hemorrhage	3	4.2
Finger clubbing	7	10
Anemia (HB 8-11 gm/l)	53	74
Leucocytosis (WBC>12000)	17	23
ESR (50-100)	34	74
ESR (> 100)	25	23
Microscopic haematuria	24	33.3
Convulsion	1	1.38
Neck stiffness	1	1.38
Vegetation(ECHO)	51	70.83

(MS) Mitral stenosis, (MR) Mitral regurgitation, (AS) aortic stenosis, (AR) Aortic regurge, (TS) tricuspid stenosis, (TR) tricuspid regurge, (PS) pulmonary stenosis, (PR) pulmonary regurge, (VSD) Ventricular septal defect, (MVP) mitral valve prolalpse.

Thirty-eight (53.3%) of the patients had apPast history of RHD and 2 (2.8%) of the patients had previous valve replacement while 1 (1.4%) had post mitral balloon dilatation for Mitral stenosis (MS). One of the patients (1.4%) had a history of previous (IE) and 26 (36%) had no history of previous cardiac disease.

Cardiac lesions on admission were mitral regurgitation (MR), which was the commonest lesion occurring in 54 (82.1%), followed by aortic regurgitation (AR) 45(63%. MR with AR was the commonest combined lesions 38(52.7%).

Other clinical signs which were detected on examination were tachycardia in the range of 90 - 120 beats/min; spleniomegaly in 11 (15.4%), hepatosplenomegaly in 3 (4.2%) and hepato-megaly^22^ (31%).

Peripheral signs like splinter hemorrhage were seen in 3 (4.2%), clubbing of the finger in 7 (10%); Janeway lesion and Osler’s node were not seen. Fiftythree (74%) of the admitted cases were anemic with hemoglobin (Hb) ranging from 8-11gm/dL. Total WBC above 12000 was seen in 17 (23%). ESR was high in the range of 50-100 in 34 (74 %), above 100 in 25 (23%) and in the range of 20-50 in 22 (30%).

Microscopic haematuria was noted in 24 patients (33.3%.Transthoracic echocardiography confirmed most of the cardiac lesions observed on clinical examination. Two cases of MR appeared to have been due to Mitral valve prolapse. Transthoracic Echo was able to detect vegetation in 51 patients (70.83%), 36 (70.58%) on Mitral cusps; 13 (25.49%) on aortic cusps, One (1.4%) on pulmonary valve, one (1.4%) on the ventricular sepal defect (VSD) border toward right ventricle.

Serum creatinine was within normal range in 64 (89%) and it was elevated in 8(11%) and 2 of them underwent dialysis and 57 (79%) of the liver enzyme was normal.

Blood culture and serology results are shown in Table 3

**Table 3:** Result of blood culture and serology

Microorganism	No.	%
Staphylococcus aureus	3	4.28
Streptococcus Viridans	2	2.85
Serology for brucellosis	2	2.85
Negative culture	65	90.27
**Total**	**72**	**100**

Revised Dukes criteria were used for the diagnosis of IE. 12 patients (16.7%) were classified as definite and 58 patients (80.5%) as possible IE. Only 2 (2.8%) cases did not fulfill the Dukes criteria but could not be rejected and they were treated as IE because of high clinical suspicion.

After adding high ESR as a minor criteria the number of patients with definite IE rose up from 12 to 37 (51.4%) and those with possible IE decreased from 58 to 35 (48.6%) patients. No patient was classified as rejected.

Forty-nine patients (68.05%) received benzyl penicillin and Gentamicin for the first two weeks and then penicillin alone for two weeks. Twenty-one patients (29.17%) were given Ceftriaxon once a day for 4 weeks; two Brucella cases (2.8%) were treated with Ceftriaxon for 6 weeks, Gentamicin for 2 weeks, doxycyclin, and Rifampicin for 12 weeks. Three patients with positive blood culture received Vancomycin instead of the standard penicillin + Gentamicin or Ceftriaxon.

Therapy extended to 8 Weeks for one patient who presented with renal failure and dialyzed before Ceftriaxon therapy was started. The renal function test completely normalized after SBE therapy (Antibiotic dose was adjusted according to creatinine clearance). Two patients were sent for valve replacement after 2 weeks of empirical therapy when clinical deterioration and worsening heart failure was seen.

## Discussion

In developed countries, the epidemiological characteristics of lE have changed over the past few decades. This shift is attributed to a number of factors including increased patient longevity and concomitant rise of degenerative valvular lesion, a marked reduction in the incidence of acute rheumatic fever and more frequent use of invasive central vascular catheters associated with an increase nosocomially acquired endocarditis^2^.

The mean age of a patient with endocarditis from western countries in the pre-antibiotic era was about 35 years and in subsequent decades the age at presentation has gradually increased to more than 50^1^,^5^,^6^.

In our patients the mean age was 28.5 years which reflects the effect of rheumatic fever and its sequelae on the heart and to uncorrected congenital heart disease. In India and Pakistan, the same young mean age is also seen, 25 and 29 years respectively^3^,^7^.

In this study women (58%) were affected more than men (42%). In contrast to the series of endocarditis from the pre-antibiotics era which did not describe any difference in gender prevalence^8^,^9^,^10^. Unlike western countries RHD was the commonest underlying heart disease in this study as indicated by a positive past history in 53.3%. Other studies from this part of the world support this finding. RHD is no longer the commonest underlying heart disease in western countries and the incidence of rheumatic fever has decreased^29^. Mitral valve prolapse (MVP) has been reported as a common underlying heart lesion in western studies^30^,^1^ but constituted only a small fraction in our series (2.8%). There was no history of intravenous drugs abuse in our patients.

Fever was the most prevalent symptom among our patients (81.9%) although it was not mentioned in (18.1%) of cases. Thus the lack of fever should not exclude the diagnosis of IE in a patient with suggestive clinical features^31^,^32^.

The other various symptoms and signs were comparable with some exceptions. Systemic embolization and peripheral signs were low. Hepatomegaly was found solely in our study which could be attributed to right sided heart failure. In addition a few patients presented with epilepsy and meningitis. These clinical presentations were not reported before.

Transthoracic echocardiography (TTE) showed that more than one valve was affected in the majority of cases. Mitral valve regurgitation was the predominant lesion in (82.1%) with female predominance. The same observation was reported in Turkey where females represented 62.5% of the IE patients^13^.

TTE was able to detect vegetations in (70.83%) of our patients which was within the range of other studies (50-93.8%)^13^,^14^. The higher range reported was due to transesophageal echocardiography study which was not available in our hospital at the time of this study. Anemia was present in (74%) which may suggest delay in suspecting diagnosis, the same results were reported in other studies^11^,^14^,^15^.

In this study, all patients had an elevated ESR which was in line with results described in other studies^14^,^23^. These observations support our opinion to add elevated ESR as minor criteria for the revised Dukes Criteria. The same proposal was also suggested in previous literature^24^. Adding high ESR as minor criteria in our study improved the diagnosis of definite IE and decreased possible IE. This improvement in the diagnosis was statistically significant (p-value .000) (Table 4).

**Table 4:** Statistical signifance of adding raised ESR at minor criteria.

			After		
			Possiple	Definate	Total
Before	Possiple	Count	35	35	60
		% within Before	58.3%	41.7%	100.0%
		% within After	100.0%	67.6%	83.3%
	Definite	Count	0	12	12
		% within Before	.0%	100.0%	100.0%
		% within After	.0%	32.4%	16.7%
Total		Count	35	37	72
		% within Before	48.6	51.4	100.0%
		% within After	100.0%	100.0%	100.0%

**Table:** Chi-Square Tests

	Value	P-value
McNemar Test		.000^a^
N of Valid Cases	72	

a Binominal distribution used.

High ESR is important in countries like our country where the use of blood culture in the diagnosis of IE is limited. Positive blood culture and serology in our patients were (9.6%) and it was markedly lower than a report from a review of 206 cases of IE seen over a 15 years period at a New York hospital which was (90%)^16^ and even lower than other reports from developing countries; Argentina (89.9%)^27^ and Tunisia (46.4%)^28^. This low result may be attributed to previous use of antibiotics, either self administration or prescribed by general practitioners who had low index of suspicion of the possibility of IE.

The diagnosis of IE is usually based on constellation of clinical findings rather than upon a single definite test result. .The Duke Criteria (Table 1) is valuable in assisting clinicians to diagnose IE. In 1994 investigators from Duke University modified the old Von Reyn criteria to include the role of echocardiography in the diagnosis. Multiple studies have validated the Dukes Criteria and compared its utility to Von Reyn criteria^12^,^18^–^21^. Each of these reports evaluated patients in whom the diagnosis of IE was established. When applying the Dukes criteria to validate our cases, 12 patients were diagnosed as definite IE and 58 patients were diagnosed as possible IE. Adding a high ESR as minor criteria, the diagnosis is improved (Table 4) hence confirming the value of these criteria in the clinical diagnosis of IE. Patients with rheumatic or congenital heart disease and high ESR need especial effort to exclude IE.

In our daily practice, there is a limited role of bacterial culture in guiding the selection of antibiotics on different clinical circumstances. Multiple factors such as low experience in specimen collection and lack of optimal conventional culture techniques may play a role in this low incidence of positive blood culture and that is why we start empirical therapy as suggested in the literature^22^.

Although all cases in this study were subjected to 3 blood cultures only seven of them were able to identify the type of organism and the suitable antibiotic and according to the results, treatment has been adjusted. In two cases serology for Brucella was positive and both of them did not develop heart failure or embolization during hospitalization. They were treated successfully with prolonged course of antibiotics^33^. Sixty five (90.4%) were unexpectedly negative cultures. These patients were given antibiotic therapy for 4-6 weeks according to the recommendation of the American Heart Association (AHA)^22^ and showed good clinical response. Two patients deteriorated with worsening heart failure and were sent for valve replacement. One patient with severe aortic stenosis was also sent for valve replacement but he died while waiting for operation.

A significant number (87.5%) of our patients were discharged in good general condition., This good result is explained by strict adherence to modified Duke Criteria in the diagnosis and following empirical antibiotic therapy as suggested in literature^22^.

Heart failure (HF) was the most common complication in our study (37.5%) and it was more severe in the patients who died. A higher incidence of HF was reported in India (41.9%)^15^ and Tunisia (45.4%)^28^. Those who died (6/9) presented with advanced heart failure due to more than one valve affection with marked left ventricle dilatation and below 40% ejection fraction.

The second complication was neurological (11.1%) which is lower than the results obtained in Tunisia (20.2%)^28^, cerebral embolization was the major cause of neurological complications and this was also seen in previous studies^14^.

In hospital mortality was (12.5%) which is lower than other reports in underdeveloped countries in Asia^7^,^24^ and comparable to the mortality in Poland^25^. The predictors of mortality in our study were high mean age and heart failure as well as intracranial hemorrhage and renal failure (Table 5). The same findings were noted in other studies^26^. A low fatality rate may be explained by low mean age of the patients who are expected to be free from other end organ damage and low virulence infection like streptococcus viridans.

**Table 5:** Comparison of patients with IE who died and those recovered.

	Died patients (No.)	Recovered patients (No.)	P.value
No. of patients	9	63	0.0001
Mean age	42.89+/-13.34	26.51+/-13.55	0.000
Predisposing RHD	3	40	0.084
**Complications**			
- Neurological	1	7	0.893
- Heart failure	6	21	0.050
- Renal failure	1	1	0.104
**Site of vegetations**			
- Mitral valve	3	33	0.285
- Aortic valve	2	11	0.728
- Pulmonary valve	0	1	0.703
- VSD	0	1	0.703
**Positive culture**		0.032	
- Streptococci viridans	0	2	
- Staphylococci Areus	2	1	
- Brucella		2	
**Treatment**			
Ceftriaxon	3	20	0.924
Pencillin+Gentamicine	6	43	
Definite IE	0	12	0.061
Probable IE	9	51	0.061

A comparison of mean age, underlying heart disease, blood cultures and treatment between those who died and those who recovered is shown in Table 5.

## Conclusion

IE in Yemen is different from the developed countries but quite similar to that reported from developing countries. It occurs in relative younger population. Rheumatic heart disease is the most important precipitating etiology. IE in MVP, degenerative heart disease and intravenous drug abusers is uncommon in Yemen. Extra effort is needed to recognize and manage streptococcal infection. General practitioners should be advised to follow the guidelines in antibiotic prophylaxis for infective endocarditis prevention.

High ESR as additional minor criteria for Dukes Criteria improves the diagnostic possibility for IE. We find that empirical antibiotic therapy, which has been suggested in the literature, is extremely valuable in our situation to decrease morbidity and mortality of IE.
